# The Defeat of the Bill

**Published:** 1894-01

**Authors:** 


### *EDITORIAL.*

The office of THE JOURNAL is in room 330 Equitable Building.

Address all communications, and make all remittances payable to THE ATLANTA MEDICAL AND SURGICAL JOURNAL, Atlanta, Ga.

Articles for publication should reach this office not later than the 15th instant.

The editors are not responsible for views expressed by contributors.

### THE DEFEAT OF THE BILL.

Our readers have learned, most of them with regret, no doubt, that the bill for the Board of Examiners was lost in the House. It was early apparent that the bill as originally introduced could not be carried, and the vote occurred on the amendment providing for three separate boards. While the friends of the measure would have much preferred the original bill, they would have accepted the substitute as a step, at least, in the right direction. The objection to the original bill which the members seemed to find it impossible to get over, namely, that it gave too much power to the regular school, of course had no force as applied to the substitute, and there was every reason to believe that the latter would meet with favorable consideration. Upon the vote, however, while there was a majority of about twenty in favor of the amended bill, the requisite number of votes was not obtained and the bill was lost by default. We cannot but regard the result as a great misfortune to the large number of people who are at the mercy of incompetent practitioners or the victims of the hordes of quacks that infest our State. The importance of this matter has not been exaggerated, and it would seem that the intelligence of the Georgia legislature ought to have been able to grasp the idea that such a measure is now a *necessary civil protection.* After two-thirds of the States of the Union have provided themselves with this protection, it would seem that our own State would not be slow in doing the same thing when one result of our negligence is to make our State an asylum for those who are not allowed to practice elsewhere.

The friends of this measure have been greatly disappointed in the legislature. But the most surprising fact of all, and one which we record with much regret, is that the bill's worst enemies in the House were *two regular physicians.* It was they who killed the bill when the respectable medical profession of the State, by their representatives, were before the legislature asking for its passage. Shame on them, and shame on the legislature that was willing to accept the dictum of these two gentlemen as opposed to the petition of the profession at large and the wishes and best interests of the people. Before the next meeting of the General Assembly we hope that these medical representatives will either have experienced a change of heart or have been retired by their constituents to the quietude of their country practice where they can doubtless serve their State to better advantage.

But we wish right here to serve notice on the Georgia legislature that they have "scotched the snake, not killed it." This matter will be brought before every successive Assembly until it becomes a law. That the friends of this measure will yet witness the success of their efforts we haven't the shadow of a doubt.

